# Trial Analysis of the Relationship between Taste and Biological Information Obtained While Eating Strawberries for Sensory Evaluation

**DOI:** 10.3390/s22239496

**Published:** 2022-12-05

**Authors:** Keisuke Maeda, Ren Togo, Takahiro Ogawa, Shin-ichi Adachi, Fumiaki Yoshizawa, Miki Haseyama

**Affiliations:** 1 Faculty of Information Science and Technology, Hokkaido University, N-14, W-9, Kita-ku, Sapporo 060-0814, Japan; 2 Center for Bioscience Research and Education, Utsunomiya University, 350, Mine-machi, Utsunomiya 321-8505, Japan; 3 Faculty of Health Sciences for Welfare, Kansai University of Welfare Sciences, 3-11-1, Asahigaoka, Kashiwabara, Osaka 582-0026, Japan; 4 School of Agriculture, Utsunomiya University, 350, Mine-machi, Utsunomiya 321-8505, Japan

**Keywords:** strawberry, biological information, brain activity, canonical correlation analysis, sensory evaluation

## Abstract

This paper presents a trial analysis of the relationship between taste and biological information obtained while eating strawberries (for a sensory evaluation). This study used the visual analog scale (VAS); we collected questionnaires used in previous studies and human brain activity obtained while eating strawberries. In our analysis, we assumed that brain activity is highly correlated with taste. Then, the relationships between brain activity and other data, such as VAS and questionnaires, could be analyzed through a canonical correlation analysis, which is a multivariate analysis. Through an analysis of brain activity, the potential relationship with "taste" (that is not revealed by the initial simple correlation analysis) can be discovered. This is the main contribution of this study. In the experiments, we discovered the potential relationship between cultural factors (in the questionnaires) and taste. We also found a strong relationship between taste and individual information. In particular, the analysis of cross-loading between brain activity and individual information suggests that acidity and the sugar-to-acid ratio are related to taste.

## 1. Introduction

Strawberries were first bred on Dutch farms in the 18th century and quickly spread worldwide. Currently, over nine million tons of strawberries are grown globally every year [1]. Since strawberries are used as fresh fruits and processed foods, various cultivars and methods of cultivation have been developed [2]. Thus, various studies have been conducted. For example, researchers have studied methods to improve cultivars [3], as well as cultivate [4], preserve [5,6], and process them [7,8].

Recently, studies on taste have received significant attention [1,9,10]. Ikegaya et al. [11] clarified the effects of sugar and organic acids (the main components in strawberry fruit juice) on the taste of strawberries. It has also been reported that the palatability of fruit is influenced by several factors, such as size, color, flavor, and texture [12,13,14]. Using multiple types of data is important for sensory evaluations. However, collecting the biological data of humans (related to texture and flavor) is difficult due to heavy burdens. To the best of our knowledge, no research has been conducted on the sensory evaluation of strawberries using heterogeneous data, including sugar and organic acid content, taste, the sizes of strawberries, and human perceptions.

Recent advances in the Internet of Things technologies have remarkably "transcended" traditional environmental sensing [15,16]. Various sensors can easily be used; we can also obtain biological data from humans. Thus, many studies using sensors have been conducted. In studies using wearable sensors, healthcare monitoring systems using vital data, such as blood pressure, heart rate, weight, and blood glucose, have been proposed [17,18]. Additionally, several studies estimated the interests of content involving human behavior [19,20] and personalized saliency (and its prediction) using gaze data [21,22,23]. Furthermore, the analysis of behavior, gaze data, and brain activity contribute to the solutions to several tasks, such as brain decoding [24,25,26,27] and certain applications [28,29,30,31,32]. Onuma et al. [31,32] showed that human brain activity calculated from functional near-infrared spectroscopy (fNIRS) is related to the taste of food [31]. fNIRS is a useful and non-invasive technique for brain imaging that measures blood flow in neural regions near the cortical surface. Minematsu et al. also showed that pleasant and unpleasant tasting foods tended to elicit decreased and increased oxyhemoglobin levels by monitoring changes in oxygenated hemoglobin levels in the anterior prefrontal cortex in response to the intake of hedonically different edibles in healthy adults [32]. From the above studies, it is expected that the taste of strawberries can be measured by brain activity through fNIRS.

**Figure 1:**
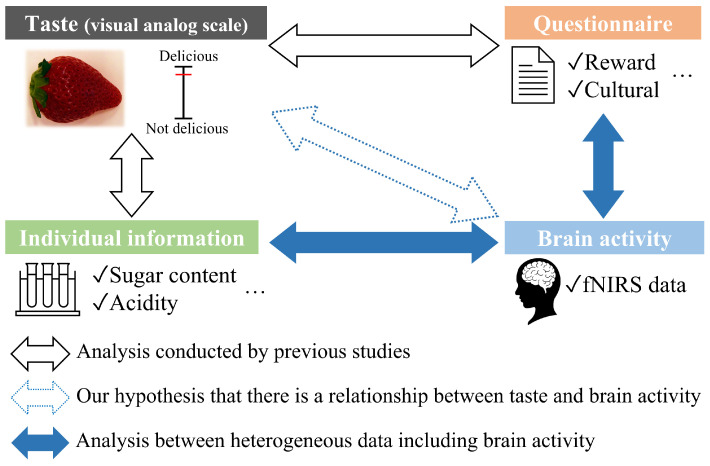
Overview of the differences between the analyses conducted in previous studies and the analysis conducted in this study. The black arrow indicates the analyses conducted by previous studies. The blue arrows and the blue arrows with broken lines indicate our hypothesis and analyses of heterogeneous data, respectively.

It is possible to analyze the taste of strawberries from a new perspective, i.e., we hypothesize that there is a relationship between brain activity and the taste of strawberries. To illustrate this advantage, Figure 1 shows an overview of the differences between the analyses conducted in previous studies and the analysis that this paper focuses on. As shown by the black arrow in Figure 1, previous studies have used taste, questionnaires (e.g., reward and culture), and individual information (e.g., size and sugar content). In the analysis of the relationship between taste and questionnaires, participants filled out various questionnaires [33]. In these analyses, it is assumed that there is a correlation between the items in the questionnaire and taste. However, if the participants cannot find this correlation, it is difficult to grasp the relationship with the taste. Additionally, since individual data of strawberries consist of numerical data, such as sugar content, it is difficult to find a quantitative relationship with the visual analog scale (VAS) assigned by human senses. Based on the hypothesis represented by the blue arrow with the broken line, we analyzed the relationships between brain activity and questionnaires, and between brain activity and individual information. We expect to extract new questionnaires that have a high correlation with taste. In the same way, clarification of the existence of potential relationships between taste and individual numerical data can be expected. These relationships correlate with the blue arrow in Figure 1. Therefore, this study clarifies the potential relationships between tastes, questionnaires, and individual information using brain activity.

In this study, we conducted a trial analysis of relationships between heterogeneous data related to strawberries based on a multivariate analysis. For the preparation of the analysis, we collected four kinds of data: VAS, questionnaires, brain activity, and individual information. Several participants ate strawberries and evaluated their taste using the VAS. We measured fNIRS data from participants; participants completed questionnaires for reward and cultural factors. Individual information consisted of information about each strawberry, such as size and sugar content. Then, we performed two kinds of analyses on the relationships between the data. Previous studies focused on the relationship between taste and other data; however, in this study, we first conducted a simple correlation analysis as the first analysis, i.e., a traditional approach. In the first analysis, we analyzed the correlation between taste and three other kinds of data. Then, we identified the data that were highly correlated with taste. This analysis was similar to previous analyses conducted by researchers since it directly focused on taste and other data. The second analysis was the canonical correlation analysis (CCA) [34]; this was conducted to find the potential relationships that could not be revealed by the first simple correlation analysis. It is a method that projects different kinds of features into a common latent space so that their correlations can be maximized. Specifically, we verified the canonical correlation between brain activity and other data (taste, questionnaires, and individual information). Through the analysis of “brain activity and taste”, we clarified that they were related. Then, in the analyses of “brain activity and questionnaire data” and “brain activity and individual information”, we identified the data that were highly correlated with brain activities by calculating the cross-loading between the data. In other words, the identified data could be regarded as potentially related to the taste. Consequently, we obtained new knowledge about the analysis of the taste of strawberries via questionnaires that were potentially highly correlated with taste; we found a strong relationship between taste and individual information. Therefore, discovering the potential relationships that could not be revealed by the first simple correlation analysis is the main contribution of this study.

## 2. Procedure of Data Acquisition and Constructed Dataset

In this section, we explain the procedure of data acquisition and details of the constructed dataset.

### 2.1. Procedure of Data Acquisition

**Figure 2:**
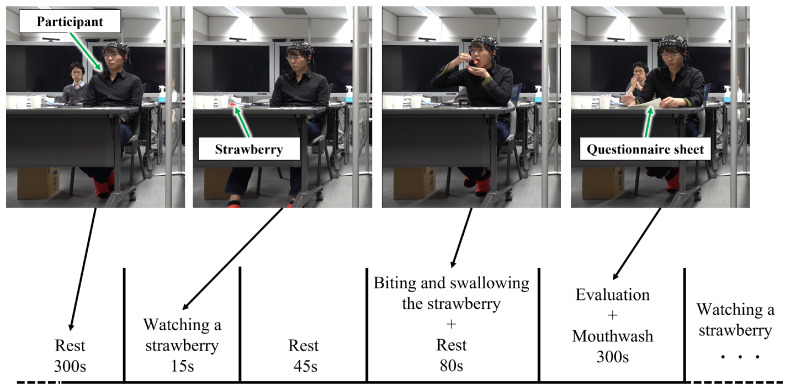
Schematic diagram of the rest and task sequences in the experiment. The signal of the temporal brain area was recorded by fNIRS during the biting and swallowing of the strawberry. After recording, evaluations of the VAS and some questionnaires were conducted. These tasks were repeated for four strawberries per participant. Note that a five-minute gap between tasks was the same condition as in the literature [37].

Nineteen participants (fourteen men and five women) participated in this experiment. The average age was 24.7 years. The minimum age of the participants was 21 years old and the maximum age was 55 years old. All participants were healthy and did not report any olfactory or gustatory disorders. Verbal and written explanations about the experiment were given to the participants and written informed consent was obtained. These experiments received approval from the Ethics Committee of Hokkaido University, Japan. Participants were seated in comfortable chairs in a room at a constant temperature (23 °C). Participants were given verbal and written instructions for the experimental procedure and fitted with the fNIRS headset. In this experiment, there were sequential blocks of rest periods and task periods, as shown in Figure 2.

During the rest periods (300 s), the participants sat on their chairs; they had their eyes opened, and were relaxed. Then, the participants watched one strawberry and its name (15 s); there was a rest period (45 s). After that, the participants took the strawberry into their mouths, bit into it, and swallowed it immediately; there was also a rest period (80 s). Finally, the participants began the evaluation of the VAS and the questionnaires, as explained in the “VAS obtained through user evaluation”. After the evaluation, participants rinsed their mouths with a cup of pure water and rested. This procedure was constructed based on previous studies [33,35]. Similar to the previous study [36], the mouthwash was performed with pure water at a constant temperature. In this study, we calculated fNIRS features, as explained in “fNIRS data”, when the participants bit and swallowed the strawberries. The above task was performed for each strawberry; thus, the participants repeated the task four times. Note that the flow of the experiments was explained to the participants. Then, in the actual experiment, the staff provided strawberries and questionnaires to the participants, and the participants performed the task. Thus, the experimental staff directly controlled the instructions. During the fNIRS measurement, the participants were filmed by a video camera, as shown in Figure 2; the fNIRS signal synchronized with the time of eating strawberries. The number of strawberries was not large, but we compensated for the small number of trials by attracting about 20 participants.

### 2.2. Dataset

This subsection explains the dataset used in the correlation analysis of heterogeneous data. We collected four kinds of data: VAS, questionnaires, brain activity, and individual information. We measured the taste of the strawberries using VAS (Section 2.2.1), and some factors related to strawberries were obtained through questionnaires (Section 2.2.2). We obtained fNIRS data from participants as brain activity (Section 2.2.3) and individual information from each strawberry (Section 2.2.4).

#### 2.2.1. VAS Obtained via User Evaluation

**Figure 3:**
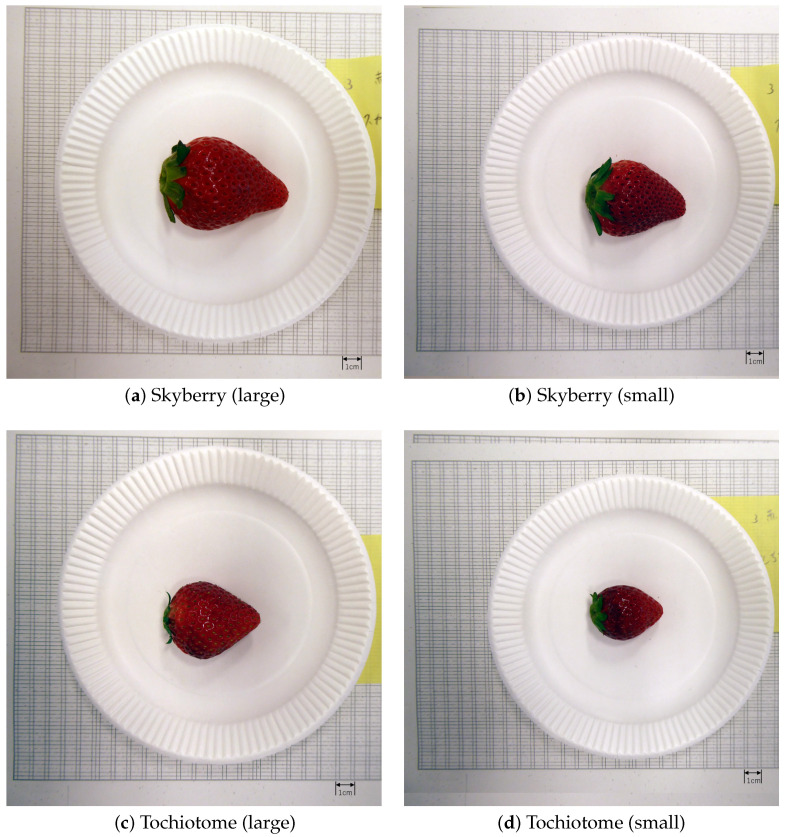
Examples of the strawberries eaten by the participants. These figures are listed as (**a**) Skyberry (large size), (**b**) Skyberry (small size), (**c**) Tochiotome (large size), and (**d**) Tochiotome (small size), respectively.

In this experiment, participants ate four strawberries and filled out two types of questionnaires about taste and other factors. Figure 3 shows examples of the strawberries eaten by the participants. In this experiment, we used two kinds of strawberries (Skyberry and Tochiotome). Skyberry and Tochiotome originated from the Tochigi prefecture, which is the most famous prefecture in Japan for strawberry cultivation. Tochiotome is a strawberry that was registered in 1996. Meanwhile, Skyberry is a new strawberry that was registered in 2014 as a successor to Tochiotome. Additionally, we adopted large-sized and small-sized strawberries to analyze the taste differences, depending on the size of the strawberry. Thus, we used four types of strawberries.

In the first questionnaire, participants evaluated the taste of the strawberries using the VAS, which is one of the most commonly used scales. They rated taste using the VAS for each strawberry. The VAS measures the intensity of pain or taste; it has been widely used in previous studies [38,39,40]. Since it is also used in the evaluation of the taste of strawberries [41,42], this index was adopted. The length of the VAS in this experiment was 100 mm. The evaluated results are presented in Table 1.

#### 2.2.2. Questionnaires Obtained via User Evaluation

Participants rated four factors related to the strawberries. Specifically, five items from each of the three factors (reward, cultural, and information factors) were selected based on the literature [33]. Additionally, a factor related to the appearance of strawberries was newly adopted. Each factor and its contents are described as follows.

1. Reward factor
  (1-1) The degree of desire caused by the addictiveness of the food;
  (1-2) The degree of level of difficulty in inhibiting urges to eat;
  (1-3) The degree of level of difficulty in inhibiting eating the food;
  (1-4) The degree of sense of satiety recognized by eating the food;
  (1-5) The degree of sense of rewarding ingredients perceived by eating the food.
2. Cultural factor
  (2-1) The degree of repeated exposure to the food;
  (2-2) The degree of dietary accustomedness to the food;
  (2-3) The degree of similarity with an accustomed food;
  (2-4) The degree of embeddedness of the food as a home-cooked taste;
  (2-5) The degree of entrenched preference for a certain food.
3. Information factor
  (3-1) The degree of visual information of the food;
  (3-2) The degree of publicity of the food;
  (3-3) The degree of health information of the food;
  (3-4) The degree of perceived safeness of the food;
  (3-5) The degree of perceived value for the price of the food.
4. Appearance factor
  (4-1) The degree of the size of the food;
  (4-2) The degree of brilliance of the food;
  (4-3) The degree of gloss of the food;
  (4-4) The degree of scent of the food.

The above 19 contents were evaluated via 19 questionnaires, as shown in Table 2. Participants evaluated them at five levels (5 (strong agreement), 4 (agreement), 3 (neutral), 2 (disagree), and 1 (strongly disagree)).

#### 2.2.3. fNIRS Data

**Figure 4:**
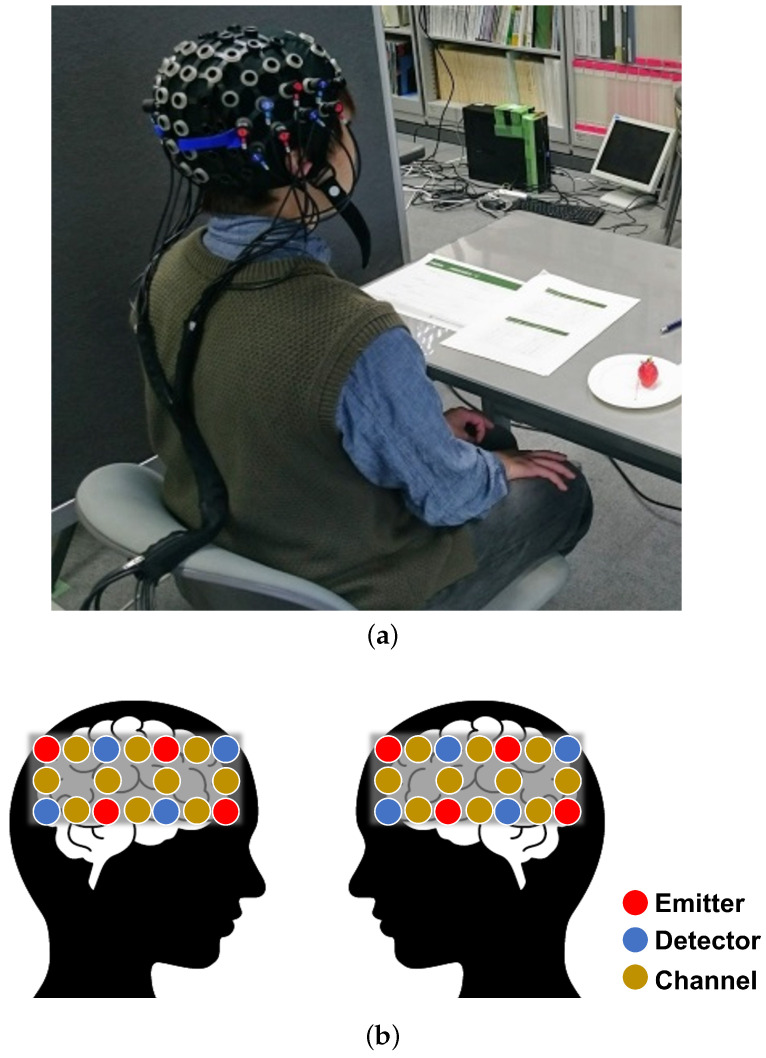
Photographic example and positions of channels. General fNIRS systems consist of (i) an emitter to illuminate a small area of tissue with light at two or more wavelengths (red and infrared range), and (ii) the detector to measure the back-scattered light emerging from the tissue. The midpoint between the emitter and detector was assumed to be the location where the change in the oxygenated and deoxygenated hemoglobin was measured; this is called the channel. (**a**) A photographic example of fNIRS data acquisition. (**b**) Positions of channels.

Light in the near-infrared region (620 to 1000 nm) of the electromagnetic spectrum penetrates most living tissues, such as bone and skin. However, hemoglobin, which is an oxygen carrier in the blood, absorbs near-infrared light. Taking advantage of this effect, fNIRS measures cortical activity by irradiating the head with near-infrared light from a light source and measuring the intensity of the light transmitted through the head with a detector. In this experiment, the temporal lobe involved in the perception of visual and gustatory stimuli was measured using LIGHTNIRS (Shimadzu Corporation Japan (http://www.shimadzu.com/, accessed on 1 November 2022)), to measure taste induced by both visual and gustatory stimuli. The measurement method was a three-wavelength absorbance calculation, which measures changes in Oxy-Hb, Deoxy-Hb, and Total-Hb. The laser wavelengths of LIGHTNIRS were 780, 805, and 830 nm. Thus, when recording fNIRS signals, we used LIGHTNIRS with 20 channels on the temporal lobe, as shown in Figure 4. We set up eight emitters and eight detectors of a near-infrared ray alternately to cover the regions in the shape of a 2 × 4 rectangle. We recorded fNIRS signals from participants and calculated five-dimensional features from each hemoglobin concentration. Specifically, we measured the average, standard deviation, root-mean-square, maximum and minimum values of the oxygenated hemoglobin, and deoxygenated hemoglobin concentrations. The time interval differs for each participant since the brain activity during the whole time of eating strawberries is targeted. In this paper, the above five types of features were calculated from each channel from the signal during the time period to avoid the influence of the time difference, and these features were calculated from each channel. Consequently, we calculated fNIRS features $f\in R^{200}$ as five-dimensional features × 20 channels × 2 (oxygenated/deoxygenated). The time interval was different for each participant because the brain activity was targeted during the entire time period of eating strawberries. In this paper, five types of features were calculated for each channel from the signals during the entire time period in order to reduce the influences of differences among participants.

#### 2.2.4. Individual Information

The individual information of strawberries was collected and converted into features. In this experiment, we collected four types of individual information: sugar content, acidity, sugar-to-acid ratio, and size (large/small). Table 3 presents the obtained individual information. This table shows that characteristics, such as the sugar content, differ depending on the type and size. Note that the strawberries used for evaluation by the participants were the same strawberries used to measure individual information.

## 3. Correlation Analysis between Heterogeneous Data

In this study, we conducted two analyses. The first analysis investigated the correlation between “taste” and “questionnaire, brain activity, and individual information” and identified the data that were highly correlated with taste. This analysis clarifies the direct relationships between the taste and other data through a simple correlation analysis. In the second analysis, we used CCA to analyze the potential correlation between “brain activity and taste”, “brain activity and questionnaire data”, and “brain activity and individual information”. Then we calculated the canonical correlation between the heterogeneous data and the cross-loading between them to identify the factors that maximize their correlations. The second analysis could discover questionnaires that were potentially highly correlated with taste; a strong relationship between taste and individual information was found.

### 3.1. Analysis of Correlation between Taste and Other Data

To identify the data that were highly correlated with taste, we calculated a correlation between the VAS representing taste and other data (questionnaires, brain activity, and individual information, such as sugar content and acidity). Before calculating the correlation, we extracted features from these data. First, from Table 1, using the VAS value $v_{p,s}$ of the strawberry $s\in \{s_{1}:\mathrm{Skyberry}\;(\mathrm{large}),\;s_{2}:\mathrm{Skyberry}\;(\mathrm{small}),\;s_{3}:\mathrm{Tochiotome}\;(\mathrm{large})\;\mathrm{and}\;s_{4}:\mathrm{Tochiotome}\;(\mathrm{small})\}$ evaluated by participant p(=1,2,...,19), we obtained features $v_{s}={\left[v_{1,s},v_{2,s},\ldots v_{19,s}\right]}^{⊤}\in R^{19}$. Then we aligned the features $v_{s}$ and obtained the VAS features $v={\left[v_{s_{1}}^{⊤},\ldots ,v_{s_{4}}^{⊤}\right]}^{⊤}\in R^{76}$.

Second, we obtained questionnaire features from questionnaires, as shown in Table 2. Specifically, using the answer value $q_{p,s}^{r}$ for questionnaire item r(∈{1,2,...,R;R being the number of questionnaires}) of the strawberry *s* evaluated by the participant *p*, we obtained features $q_{s}^{r}={\left[q_{1,s}^{r},q_{2,s}^{r},...,q_{19,s}^{r}\right]}^{⊤}\in R^{19}$. To calculate the correlation between each item and VAS in the first analysis, we aligned the features $q_{s}^{r}$ and obtained questionnaire features $q^{r}={\left[{q_{s_{1}}^{r}}^{⊤},\ldots ,{q_{s_{4}}^{r}}^{⊤}\right]}^{⊤}\in R^{76}$.

Third, as explained in the “fNIRS data”, we acquired fNIRS data from the participant *p* while eating the strawberry *s* and calculated 200-dimensional features; the elements were various statistics, such as average and standard deviation. In this analysis, we applied the principal component analysis (PCA) [43], one of the simplest dimensionality reduction methods to remove various noises included in the fNIRS data. We adopted PCA as feature preprocessing instead of data preprocessing. Consequently, low-dimensional features ${\tilde{f}}_{p,s}\in R^{d}$ were calculated. Note that we set the dimension *d* after the PCA so that the cumulative contribution rate was larger than 90%. In this analysis, the dimension *d* was set to 9. Dimensionality reduction of fNIRS features is commonly used in studies using machine learning. For example, in previous studies, i.e., [44,45,46], a local Fisher discriminant analysis [47]-based approach was adopted to perform supervised dimensionality reduction on fNIRS data. However, in [30], dimensionality reductions of fNIRS data were conducted based on PCA. Since this experiment investigated whether there was a correlation between each dimension for each feature, we calculated fNIRS features $f^{d}={\left[{\tilde{f}}_{1,s_{1}}^{d},{\tilde{f}}_{2,s_{1}}^{d},\ldots ,{\tilde{f}}_{19,s_{1}}^{d},{\tilde{f}}_{1,s_{2}}^{d},\ldots ,{\tilde{f}}_{19,s_{4}}^{d}\right]}^{⊤}\in R^{76}\;\left(d=1,2,...,9\right)$.

Finally, by employing $i_{p,s}^{u}$ for the individual information u(∈{sugar content, acidity, sugar-to-acid ratio, and size}) of the strawberry *s* eaten by the participant *p*, we obtained features $i_{s}^{u}={\left[i_{1,s}^{u},i_{2,s}^{u},...,i_{19,s}^{u}\right]}^{⊤}\in R^{19}$. Then, we aligned the features $i_{s}^{u}$ and obtained individual features $i^{u}={\left[{i_{s_{1}}^{u}}^{⊤},\ldots ,{i_{s_{4}}^{u}}^{⊤}\right]}^{⊤}\in R^{76}$. Note that when *u* = “size”, we set $i_{p,s}^{u}$ to one if the size of the strawberry was large; otherwise, $i_{p,s}^{u}$ was set to zero.

In this analysis, we calculated Pearson’s correlation coefficient, defined as follows:

(1) $$\begin{array}{cc} \eta^{ab}= & Corr(a,b)\\ = & \frac{\sum_{j=1}^{76}\left(a_{j}-\bar{a}\right)\left(b_{j}-\bar{b}\right)}{\sqrt{\sum_{j=1}^{76}{\left(a_{j}-\bar{a}\right)}^{2}}\sqrt{\sum_{j=1}^{76}{\left(b_{j}-\bar{b}\right)}^{2}}}, \end{array}$$

where a and b are features; $\bar{a}$ means the average of a. In this analysis, since the correlation between taste and other data can be calculated, we set a to v. Additionally, b was set to $q^{r}$, $f^{d}$, or $i^{u}$. Thus, we can identify data that were highly correlated with taste by searching $\eta^{vq^{r}}$, $\eta^{vf^{d}}$, and $\eta^{vi^{u}}$ in this analysis.

### 3.2. Analysis of Potential Correlation between Heterogeneous Data through CCA

In this analysis, we searched for the potential correlation between taste and brain activity based on CCA, which projects different types of features into the common latent space. In CCA, given two kinds of feature matrices $A=\left[a^{1},a^{2},...,a^{D_{a}}\right]\in R^{76\times D_{a}}$ and $B=\left[b^{1},b^{2},...,b^{D_{b}}\right]\in R^{76\times D_{b}}$, we estimated the projection vectors $\phi_{a}$ and $\phi_{b}$ that satisfy the following correlation:

(2) $$\begin{array}{c} \arg\max_{\phi_{a},\phi_{b}}\frac{\phi_{a}^{⊤}C_{AB}\phi_{b}}{\sqrt{\phi_{a}^{⊤}C_{AA}\phi_{a}}\sqrt{\phi_{b}^{⊤}C_{BB}\phi_{b}}}, \end{array}$$

where

(3) $$\begin{array}{c} C_{AB}=A^{⊤}B,\;\;\;C_{AA}=A^{⊤}A,\;\;\;C_{BB}=B^{⊤}B. \end{array}$$

To solve Equation (2), we solved the following Lagrange problem:

(4) $$\begin{array}{c} L\left(\phi_{a},\phi_{b}\right)=\;\phi_{a}^{⊤}C_{AB}\phi_{b}-\frac{\lambda_{a}}{2}\left(\phi_{a}^{⊤}C_{AA}\phi_{a}-1\right)-\frac{\lambda_{b}}{2}\left(\phi_{b}^{⊤}C_{BB}\phi_{b}-1\right), \end{array}$$

where $\lambda_{a}=\lambda_{b}\;\left(=\lambda \right)$, and λ is defined as follows. Then, we solved the following eigenvalue problems:

(5) $$\begin{array}{c} C_{AA}^{-1}C_{AB}C_{BB}^{-1}C_{AB}^{⊤}\phi_{a}=\lambda^{2}\phi_{a},\;\;\;C_{BB}^{-1}C_{AB}^{⊤}C_{AA}^{-1}C_{AB}\phi_{b}=\lambda^{2}\phi_{b}, \end{array}$$

where λ correlates to the eigenvalue of this problem. Since we can obtain multiple eigenvalues λ and their correlating eigenvectors $\phi_{a}$ and $\phi_{b}$ as solutions to the above problem, we obtain the projection matrices as $\Phi_{a}=\left[\phi_{a}^{1},\phi_{a}^{2},...,\phi_{a}^{D_{cca}}\right]\in R^{D_{a}\times D_{cca}}$ and $\Phi_{b}=\left[\phi_{b}^{1},\phi_{b}^{2},...,\phi_{b}^{D_{cca}}\right]\in R^{D_{b}\times D_{cca}}$ by aligning $D_{cca}$ eigenvectors, respectively. We can obtain the canonical features $\hat{A}=\left[{\hat{a}}^{1},{\hat{a}}^{2},\cdots ,{\hat{a}}^{D_{cca}}\right]\in R^{76\times D_{cca}}$ and $\hat{B}=\left[{\hat{b}}^{1},{\hat{b}}^{2},\cdots ,{\hat{b}}^{D_{cca}}\right]\in R^{76\times D_{cca}}$, which can consider their relationships as follows:

(6) $$\begin{array}{c} \hat{A}=A\Phi_{a},\;\;\;\hat{B}=B\Phi_{b}. \end{array}$$

In the second analysis, to reveal the relationships between brain activity and other data, we applied CCA to the correlating features. First, we reveal the existence of the relationship between taste and brain activity through the analysis between them. We used VAS features $V={\left[v_{s_{1}}^{⊤},\ldots ,v_{s_{4}}^{⊤}\right]}^{⊤}\in R^{76\times 1}$ and a brain feature matrix, as $F=\left[f^{1},f^{2},...,f^{9}\right]\in R^{76\times 9}$. Given V and F, we estimate the projection matrices $\Phi_{v}$ and $\Phi_{f_{v}}$. Consequently, we obtain canonical features $\hat{V}=\left[{\hat{v}}^{1},{\hat{v}}^{2},\cdots ,{\hat{v}}^{D_{cca}^{vf}}\right]\in R^{D_{cca}^{vf}}$ and $\hat{F_{v}}=\left[{\hat{f}}_{v}^{1},{\hat{f}}_{v}^{2},\cdots ,{\hat{f}}_{v}^{D_{cca}^{vf}}\right]$, which consider their relationships as follows:

(7) $$\begin{array}{ccc} \hat{V}= & V\Phi_{v},\;\;\;{\hat{F}}_{v}= & F_{v}\Phi_{f_{v}}. \end{array}$$

Note that the dimension of the canonical features is limited to the minimum dimensions among original features. Since dimensions of the original VAS and fNIRS features are set to 1 and 9, respectively, $D_{cca}^{vf}$ is set to 1. Finally, we measure the canonical correlation $\eta_{\mathrm{cca}}^{{\hat{v}}^{1}{\hat{f}}_{v}^{1}}=Corr\left({\hat{v}}^{1},{\hat{f}}_{v}^{1}\right)$ to verify the existence of canonical correlation between taste and brain activity.

Similar to the above motivation, we revealed the existence of relationships between “brain activity and questionnaire data” and “brain activity and individual information” using CCA. We calculated the cross-loading between them and identified the data that were highly correlated with brain activity. Thus, we discovered questionnaires that were potentially highly correlated with taste and found a strong relationship between taste and individual information. In this analysis, we calculated the canonical correlation and identified the data to maximize the canonical correlations by calculating the cross-loading between the information. Before the detailed approach for calculating the cross-loading, we defined the feature matrices of CCA. We used a questionnaire feature matrix, as $Q=\left[q^{1},q^{2},...,q^{R}\right]\in R^{76\times R}$, and a feature matrix $I=\left[i^{1},i^{2},...,i^{4}\right]\in R^{76\times 4}$ for individual information. Similar to the above analysis of taste and brain activity, we obtain the canonical features $\hat{Q}=\left[{\hat{q}}^{1},{\hat{q}}^{2},\cdots ,{\hat{q}}^{D_{cca}^{qf}}\right]\in R^{76\times D_{cca}^{qf}}$ and $\hat{F_{q}}=\left[{\hat{f}}_{q}^{1},{\hat{f}}_{q}^{2},\cdots ,{\hat{f}}_{q}^{D_{cca}^{qf}}\right]\in R^{76\times D_{cca}^{qf}}$, which consider their relationships as follows:

(8) $$\begin{array}{ccc} \hat{Q}= & Q\Phi_{q},\;\;\;{\hat{F}}_{q}= & F_{q}\Phi_{f_{q}}. \end{array}$$

In this analysis, we measured the canonical correlation $Corr({\hat{q}}^{1},{\hat{f}}_{q}^{1})$ for the first canonical feature and identified the questionnaire *r* that was highly correlated with the brain activity based on the cross-loading $Corr(q^{r},{\hat{f}}_{q}^{1})$. Furthermore, following the same procedure, we obtained the canonical features $\hat{I}=\left[{\hat{i}}^{1},{\hat{i}}^{2},\cdots ,{\hat{i}}^{D_{cca}^{if}}\right]\in R^{76\times D_{cca}^{if}}$ and $\hat{F_{i}}=\left[{\hat{f}}_{i}^{1},{\hat{f}}_{i}^{2},\cdots ,{\hat{f}}_{i}^{D_{cca}^{if}}\right]\in R^{76\times D_{cca}^{if}}$, which considered their relationships as follows:

(9) $$\begin{array}{ccc} \hat{I}= & I\Phi_{i},\;\;\;{\hat{F}}_{i}= & F_{i}\Phi_{f_{i}}. \end{array}$$

In this analysis, we measured the canonical correlation $Corr({\hat{i}}^{1},{\hat{f}}_{i}^{1})$ and identified the information *u* that was highly correlated with the brain activity based on the cross-loading $Corr(i^{u},{\hat{f}}_{i}^{1})$.

## 4. Experimental Results

### 4.1. Results of Correlation between Taste and Other Data

**Figure 5:**
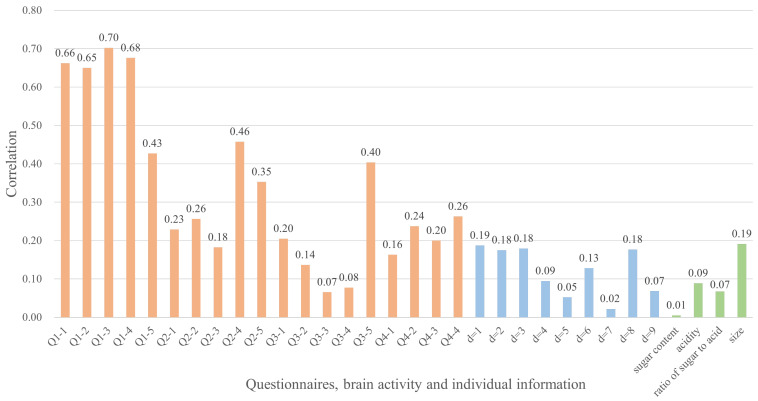
Correlation between taste and other data. The orange, blue, and green bar charts correlate with the questionnaires, brain activity, and individual information, respectively.

Figure 5 shows the results of the correlation between taste and other data. This figure shows that questionnaires had a high correlation with taste among all participants. In particular, the values of the reward factor (Q1) were high, and four of the top five items were considered as reward factors. This phenomenon of a strong correlation between the reward factor and taste has also been confirmed in a previous study [33]; thus, the results are reasonable.

Next, we focus on the correlation coefficient between brain activity and taste. Half of the features obtained from brain activity were about 0.2, but it was not clear that they were correlated. To analyze the correlation coefficients in more detail, we calculated the *p*-value, which indicates whether the correlation coefficients are significant or not. The *p*-values correlating to the correlation coefficients in Figure 5 are shown in Table 4. If the *p*-value is low, the correlating correlation coefficient is not zero, i.e., these data are correlated. Table 4 shows that the *p*-values of the correlation coefficients between brain activity information and taste are about 0.1 for d=1,2,3,8, confirming the existence of the correlation between these data.

In this present analysis, which focused on direct correlations, the reward factors (Q1) in the questionnaires strongly correlated with taste. A part of the brain activity was also correlated with taste. However, other data needed further verification; thus, we will analyze the potential correlation through brain activity in the next subsection.

### 4.2. Results of the Potential Correlation between Heterogeneous Data via CCA

Table 5 presents the results of the canonical correlation between heterogeneous data. As shown in this table, “feature” represents the dimension of the canonical feature, and “canonical correlation” represents the value calculated with the canonical features. The first canonical feature is projected by the eigenvector correlating to the largest eigenvalue. Additionally, the “*p*-value” indicates whether the canonical correlation is significant or not. The table shows that the canonical correlation between brain activity and taste is 0.4, and its correlating *p*-value is zero, confirming that brain activity is potentially correlated with taste. Additionally, the canonical correlations of “brain activity and questionnaires” and “brain activity and individual information” are also high, and their *p*-values are low. Therefore, there is a latent relationship between taste, questionnaires, and individual information through brain activity. Although the first analysis shows that the individual information is not related to taste due to the higher *p*-value shown in Table 4, the *p*-values of the canonical correlation between brain activity and individual information are very low. Thus, the results suggest that individual data, such as numerical data, can be correlated with taste. Furthermore, by clarifying which data from questionnaires and individual information have high canonical correlations with brain activity, it is expected that it will be possible to clarify which data are potentially correlated with taste.

**Figure 6:**
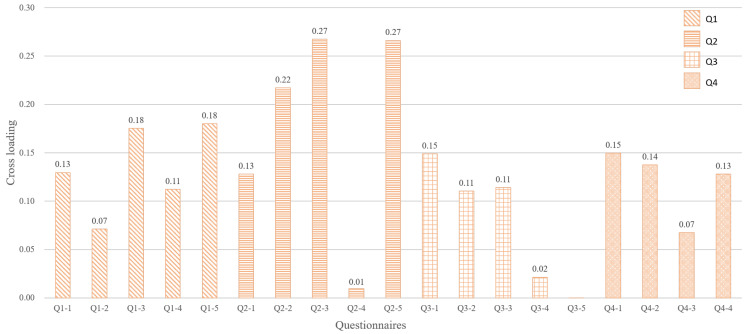
Cross-loading of “brain activity and questionnaires”.

**Figure 7:**
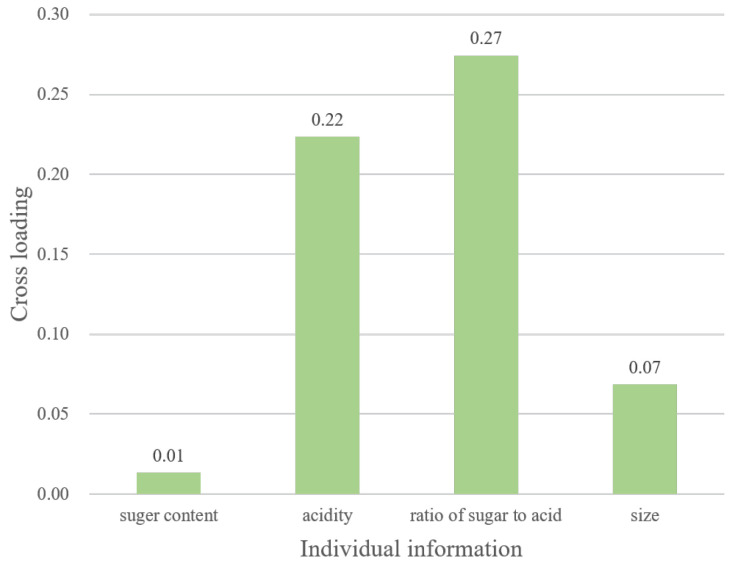
Cross-loading of “brain activity and individual information”.

Figure 6 and Figure 7 show the results of the analysis to identify the factors that are highly correlated with brain activity, which clarifies the above point. Figure 6 shows the cross-loading between brain activity and questionnaires. This figure shows that the cultural factor (Q2) is particularly correlated with brain activity. In other words, taste and cultural factors are potentially correlated. In the first analysis, th reward factor (Q1) was highly correlated with taste; however, this analysis can provide some other results. From these results, we consider that this is a discovery that can be obtained through brain activity. Furthermore, Figure 7 shows the cross-loading between brain activity and individual information. It can be seen that the sugar-to-acid ratio is correlated with brain activity. Thus, the balance between sweetness and acidity is potentially correlated with taste. Therefore, analyzing canonical correlations between heterogeneous data is effective since we can discover the potential relationships that cannot be revealed by the first simple correlation analysis.

The experimental results confirm that brain activity is potentially correlated with other data about strawberries. The above results verify that taste is likely to have a potential correlation between the cultural factor and the sugar-to-acid ratio. In other words, the estimation of the VAS representing taste becomes feasible using these data. Although it is revealed that our analysis is valid and fruitful, it has several limitations. It is well-known that brain activity often contains noise. In this study, we addressed this problem by employing PCA to calculate the fNIRS features. However, the extraction of principal components from features is likely insufficient, which is a limitation of this analysis. In future studies, we will calculate more reliable canonical correlations by applying denoising methods for fNIRS data, such as the previously reported method [45]. In the second analysis, when the canonical correlation between brain activity and the taste was calculated using CCA, the canonical features were limited to one dimension since those of the features representing taste were one dimension. Generally, there is a limit to the expressive power of one-dimensional features. Therefore, we will also increase the number of dimensions of features expressing taste by adding evaluations other than VAS and will calculate more reliable canonical correlations. As a solution to this problem, multivariate analysis models that introduce label dequantization have been proposed [48,49], and their use will be considered in future work. In addition, while general CCA is based on two types of data, methods based on multi-view CCA are effective when various types of data are used [50,51], as in our experiment in this paper. Capturing more complex correlations will become feasible by introducing the above methods.

## 5. Conclusions

In this paper, we conducted a trial analysis of the relationship between taste and biological information obtained while eating strawberries (for a sensory evaluation). We collected four types of data: taste, questionnaire, brain activity, and individual information. We also compared their potential correlations based on CCA. The experiments suggested that brain activity data were associated with information about taste. Furthermore, it was revealed that the information about food culture and the sugar-to-acid ratio was correlated with brain activity. This confirms that the cultural factors in questionnaires are potentially highly correlated with taste, and there is a strong relationship between taste and individual information. Therefore, these results can lead to the construction of a machine learning model for estimating taste using heterogeneous data.

## Figures and Tables

**Table 1:** The VAS, which is a score in millimeters of each strawberry evaluated by each participant. “Ave” and “Std” mean the average and standard deviations of the VAS, respectively.

ID	Skyberry		Tochiotome	
	(Large)	(Small)	(Large)	(Small)
1	88	77	93	89
2	84	10	63	56
3	79	59	89	81
4	75	78	88	74
5	73	69	76	77
6	83	76	6	6
7	70	70	82	80
8	74	86	61	56
9	80	81	74	68
10	52	39	64	71
11	88	69	96	96
12	50	70	74	74
13	41	18	29	11
14	58	55	52	48
15	71	60	83	53
16	83	73	91	80
17	57	53	68	47
18	76	55	48	42
19	48	64	67	65
Ave	70	61	69	62
Std	15	20	23	24

**Table 2:** The 19 questionnaires for the 4 factors.

Q1	Reward factor
Q1-1	Does it taste like a habit?
Q1-2	Does it taste like something you would want to pick up?
Q1-3	Does one bite make you want to take another bite?
Q1-4	Are you satisfied with the taste?
Q1-5	Do you think that the food tastes good
	because of its fatty sweetness and delicious taste?
Q2	Cultural factor
Q2-1	Does it taste familiar to you?
Q2-2	Have you ever eaten food that tasted the same or similar to you?
Q2-3	Have you eaten food like this many times?
Q2-4	Do you think your family will like the taste of this food?
Q2-5	Did you like the taste since you were a child?
Q3	Information factor
Q3-1	Does the food look good to you?
Q3-2	Have you seen this food in advertisements
	or heard about it by word of mouth?
Q3-3	Have you ever heard of the health benefits?
Q3-4	Do you feel comfortable with the ingredients?
Q3-5	Do you think it looks expensive?
Q4	Appearance factor
Q4-1	Is the size of this food desirable?
Q4-2	Does it look colorful and delicious?
Q4-3	Does it look shiny and delicious?
Q4-4	Do you think it smells good?

**Table 3:** Sugar content, acidity, and sugar-to-acid ratio of the strawberries.

ID	Sugar Content(%)				Acidity (%)				Sugar to Acid Ratio			
	Skyberry		Tochiotome		Skyberry		Tochiotome		Skyberry		Tochiotome	
	Large	Small	Large	Small	Large	Small	Large	Small	Large	Small	Large	Small
1	8.3	9.7	14.7	10.5	0.76	0.71	0.72	0.61	10.9	13.7	20.4	17.2
2	7.7	11.1	9.0	9.5	0.76	0.57	0.60	0.61	10.1	19.5	15.0	15.6
3	10.0	9.0	8.8	11.5	0.98	0.58	0.66	0.60	10.2	15.5	13.3	19.2
4	10.2	10.8	12.7	12.0	0.66	0.62	0.77	0.63	15.5	17.4	16.5	19.0
5	8.9	11.7	9.7	9.6	0.59	0.56	0.65	0.71	15.1	20.9	14.9	13.5
6	9.9	10.8	13.4	10.8	0.64	0.70	0.68	0.70	15.5	15.4	19.7	15.4
7	9.7	10.3	10.8	11.3	0.58	0.64	0.67	0.62	16.7	16.1	16.1	18.2
8	9.2	9.3	9.4	8.4	0.54	0.63	0.52	0.82	17.0	14.8	18.1	10.2
9	9.1	9.0	9.9	9.1	0.58	0.58	0.72	0.58	15.7	15.5	13.8	15.7
10	8.7	9.2	11.6	10.6	0.59	0.57	0.82	0.58	14.7	16.1	14.1	18.3
11	10.1	9.5	9.7	8.6	0.58	0.54	0.48	0.52	17.4	17.6	20.2	16.5
12	9.3	10.0	10.8	10.2	0.50	0.59	0.72	0.55	18.6	16.9	15.0	18.5
13	8.5	9.2	10.4	9.4	0.57	0.74	0.64	0.59	14.9	12.4	16.3	15.9
14	10.2	9.7	11.8	11.7	0.51	0.50	0.66	0.54	20.0	19.4	17.9	21.7
15	8.5	10.4	10.0	8.4	0.57	0.51	0.58	0.88	14.9	20.4	17.2	9.5
16	10.4	7.7	11.4	10.4	0.78	0.90	0.78	0.69	13.3	8.6	14.6	15.1
17	9.3	10.1	10.8	8.3	0.56	0.70	0.72	0.63	16.6	14.4	15.0	13.2
18	9.1	7.0	10.4	9.4	0.68	0.47	0.67	0.60	13.4	14.9	15.5	15.7
19	9.1	8.7	9.5	10.9	0.75	0.60	0.63	0.63	12.1	14.5	15.1	17.3
Ave	9.27	9.64	10.8	10.0	0.64	0.62	0.67	0.64	14.9	16.0	16.2	16.1
Std	0.72	1.11	1.50	1.15	0.12	0.10	0.08	0.09	2.63	2.85	2.08	2.93

**Table 4:** *p*-value for correlation between taste and other data.

Data		*p*-Value
Questionnaires	Q1-1	0.000
	Q1-2	0.000
	Q1-3	0.000
	Q1-4	0.000
	Q1-5	0.000
	Q2-1	0.047
	Q2-2	0.025
	Q2-3	0.115
	Q2-4	0.000
	Q2-5	0.002
	Q3-1	0.076
	Q3-2	0.243
	Q3-3	0.575
	Q3-4	0.507
	Q3-5	0.000
	Q4-1	0.159
	Q4-2	0.039
	Q4-3	0.084
	Q4-4	0.022
Brain activity	d=1	0.105
	d=2	0.130
	d=3	0.122
	d=4	0.416
	d=5	0.655
	d=6	0.271
	d=7	0.855
	d=8	0.127
	d=9	0.558
Individual information	sugar content	0.965
	acidity	0.446
	sugar-to-acid ratio	0.565
	size	0.098

**Table 5:** Canonical correlation between heterogeneous data.

Data	Canonical	Canonical	*p*-Value
	Feature	Correlation	
“Brain activity and taste”	1st	0.40	0.000
“Brain activity and questionnaires”	1st	0.81	0.000
	2nd	0.75	0.000
	3rd	0.68	0.000
	4th	0.59	0.000
	5th	0.58	0.000
	6th	0.52	0.000
	7th	0.47	0.000
	8th	0.42	0.000
	9th	0.21	0.073
“Brain activity and individual information”	1st	0.53	0.000
	2nd	0.41	0.000
	3rd	0.31	0.007
	4th	0.15	0.187

## Data Availability

The dataset is not available.
